# Flow Diversion of a Thrombosed Middle Cerebral Artery Bifurcation Aneurysm Presenting With Ischemic Stroke: A Case Report

**DOI:** 10.7759/cureus.98718

**Published:** 2025-12-08

**Authors:** Chibueze I Agwu, Rami Z Morsi, Archit B Baskaran, Michael C Hurley

**Affiliations:** 1 Pritzker School of Medicine, University of Chicago, Chicago, USA; 2 Neurology, University of Chicago Medicine, Chicago, USA; 3 Radiology, University of Chicago Medicine, Chicago, USA

**Keywords:** bifurcation aneurysm, clot destabilization, flow diversion, interventional neurosurgery, thrombosed middle cerebral artery

## Abstract

Middle cerebral artery (MCA) bifurcation aneurysms typically present incidentally on imaging or with subarachnoid hemorrhage and are often managed surgically due to their peripheral location and complex anatomy. Ischemic stroke due to spontaneous Intrasaccular thrombosis is extremely rare and poses a management dilemma due to the combined risk of further thromboembolism and aneurysm rupture.

A 68-year-old woman with a history of smoking presented with symptoms of a left MCA syndrome with acute left MCA territory infarcts on magnetic resonance imaging (MRI). Computed tomography angiography demonstrated a left MCA bifurcation aneurysm with an atypical luminal contour, which suggested intrasaccular thrombus. Vessel wall MRI supported the diagnosis of intrasaccular thrombus and aneurysm instability. In view of the risks of both recurrent thromboembolism and rupture, endovascular flow diversion was performed across the aneurysm into the superior division with initiation of dual antiplatelet therapy. The procedure was well tolerated and yielded excellent angiographic results.

The management of partially thrombosed MCA aneurysms presenting with ischemic stroke remains challenging and is not well defined. Although flow diversion addressed both the thromboembolic and rupture risks in this case, it carries a significant risk of iatrogenic clot destabilization. Broader data collection through multicenter registries may help clarify the natural history and optimal management of this rare presentation.

## Introduction

Middle cerebral artery (MCA) bifurcation aneurysms account for 18-36% of all intracranial aneurysms [1]. Large series estimate an overall annual rupture risk of approximately 0.36%, with small incidental aneurysms (e.g., <7 mm anterior circulation aneurysms, including MCA bifurcation, in the ISUIA cohort) carrying particularly low rupture rates [2]. Aside from subarachnoid hemorrhage, aneurysms in the cavernous region and basal cisterns may occasionally cause cranial neuropathies, for example, a posterior communicating artery aneurysm compressing the oculomotor nerve.

Thromboembolic stroke is an exceptionally rare presentation of an intracranial aneurysm, usually associated with giant or dissecting aneurysms. In the context of the relatively high prevalence of incidental saccular aneurysms (2%-4% in the general population), ischemic stroke is far more often coincidental rather than causally related, making diagnosis of an aneurysm-related embolic source challenging [3,4]. Reports of acute intra-aneurysmal thrombosis producing cerebral infarction and subsequently managed by endovascular intervention remain exceedingly scarce.

Here, we present a rare case of an MCA bifurcation aneurysm with spontaneous intra-aneurysmal thrombosis presenting as ischemic stroke. Initial computed tomography angiography (CTA) findings were confusing, but the working diagnosis of intra-aneurysmal thrombosis was supported by vessel wall imaging and interval change on serial conventional angiography prior to and after treatment by flow diversion.

## Case presentation

A 68-year-old woman presented with acute aphasia, right facial droop, and right upper extremity numbness (National Institutes of Health Stroke Scale score of 5). She had no prior medical history, was not on medications, and was an active smoker.

**Figure 1:**
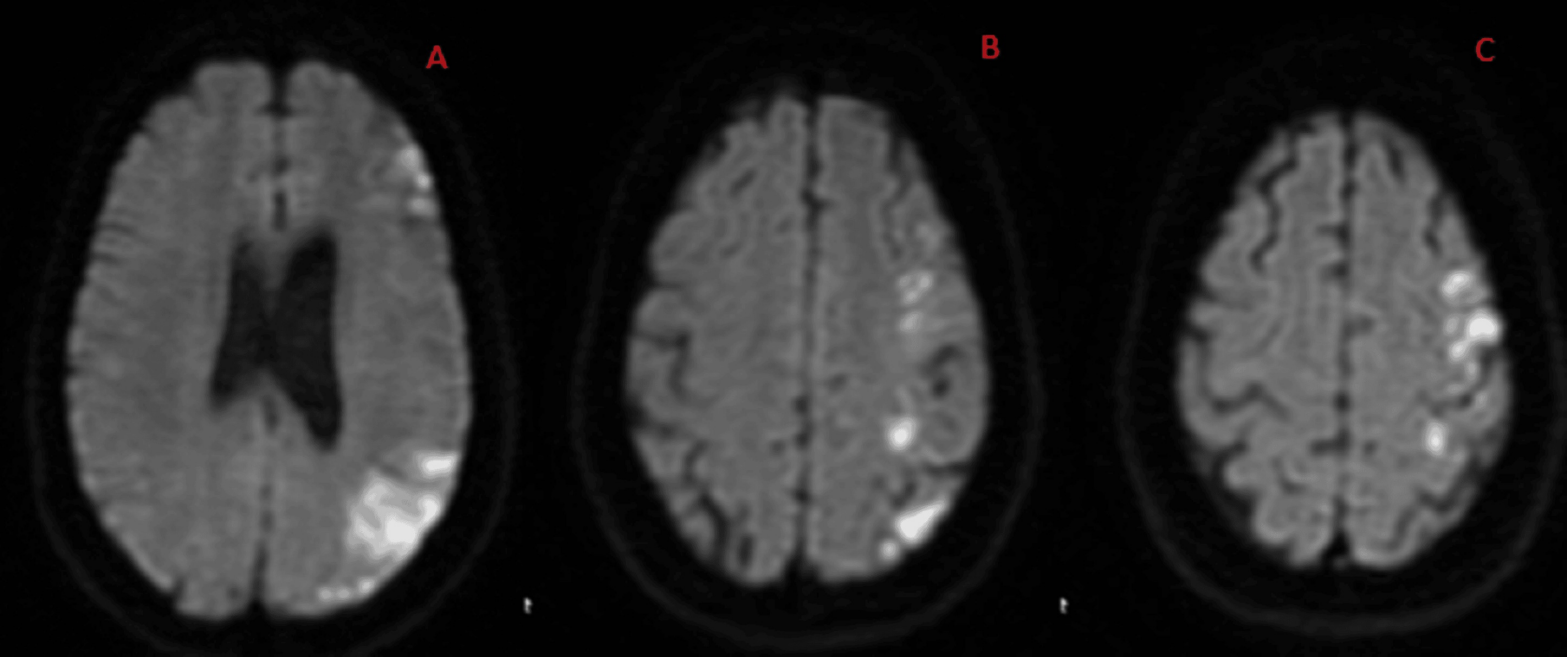
Diffusion-weighted imaging sequence demonstrating diffusion restriction hits across the left cerebral hemisphere at deep (A), middle (B), and high (C) cross-sections.

**Figure 2:**
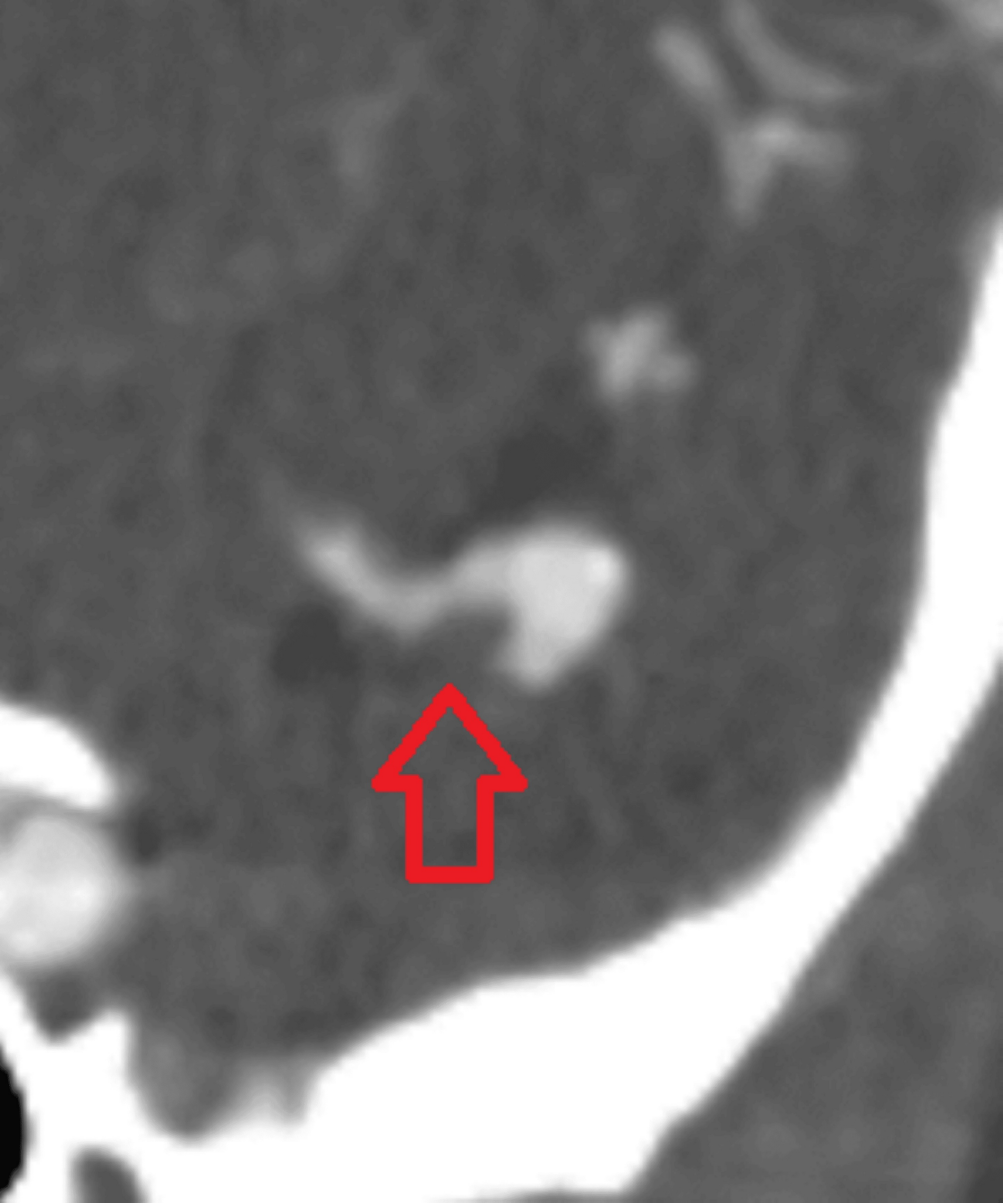
CTA demonstrating a possible filling defect in a saccular aneurysm previously thought to be a curvilinear partly fusiform-shaped aneurysm. Note the slight difference between the darker gray the arrow demarcates and the lighter gray above it. CTA: Computed tomography angiography

**Figure 3:**
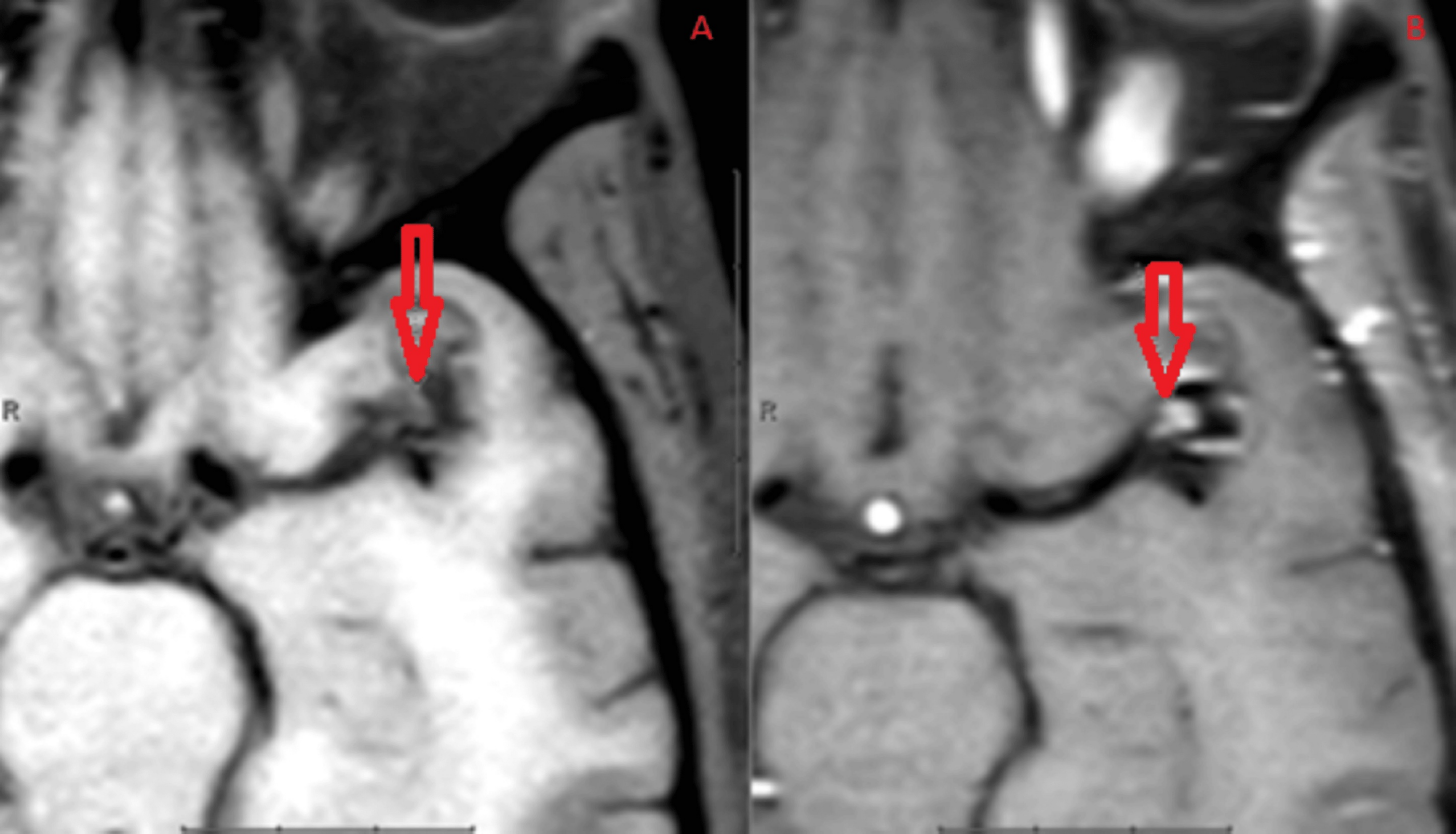
Pre (A) and post (B) T1 contrast images demonstrating contrast enhancement within the aneurysmal sac suggestive of an intra-aneurysmal filling defect.

Brain magnetic resonance imaging (MRI) demonstrated acute infarcts in the left frontal, parietal, and temporal lobes with a watershed distribution. CTA revealed mild carotid siphon atherosclerosis and several small aneurysms (3-4 mm) involving the bilateral paraclinoid internal carotid arteries (ICAs), right MCA bifurcation, and the right pericallosal anterior cerebral artery. A larger, irregular 7 mm aneurysm was identified at the left MCA bifurcation (Figure 1). Initially interpreted as partly fusiform (Figure 2), a closer review raised concern for a filling defect within a more conventionally shaped saccular aneurysm. Vessel wall MRI was obtained and supported intra-aneurysmal thrombosis (Figure 3).

**Figure 4:**
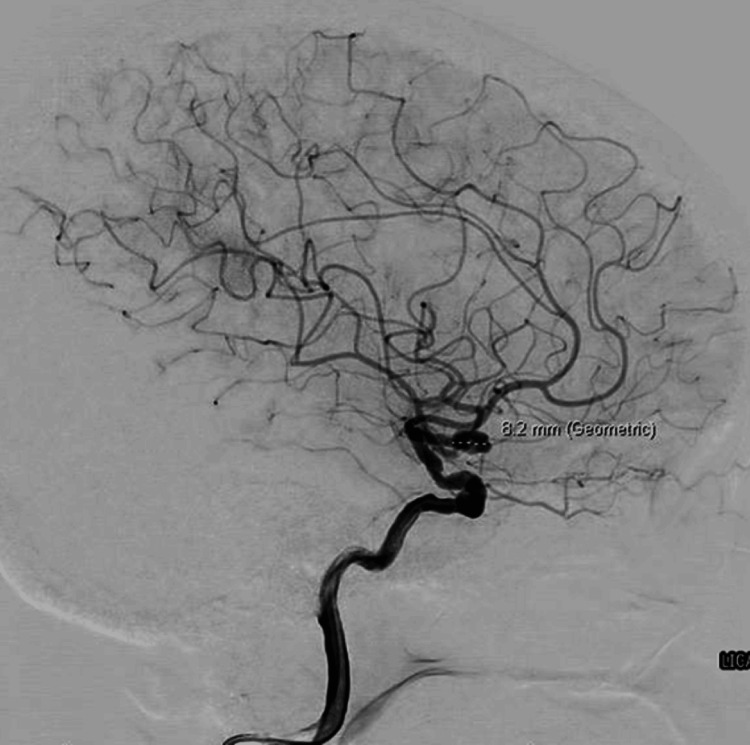
Diagnostic cerebral angiography demonstrating an 8 mm saccular left MCA bifurcation aneurysm containing a filling defect. MCA: Middle cerebral artery

Diagnostic cerebral angiography confirmed an 8 mm saccular left MCA bifurcation aneurysm containing a filling defect (Figure 4). Both MCA divisions arose from the aneurysm base, with partial attenuation of the inferior division origin.

**Figure 5:**
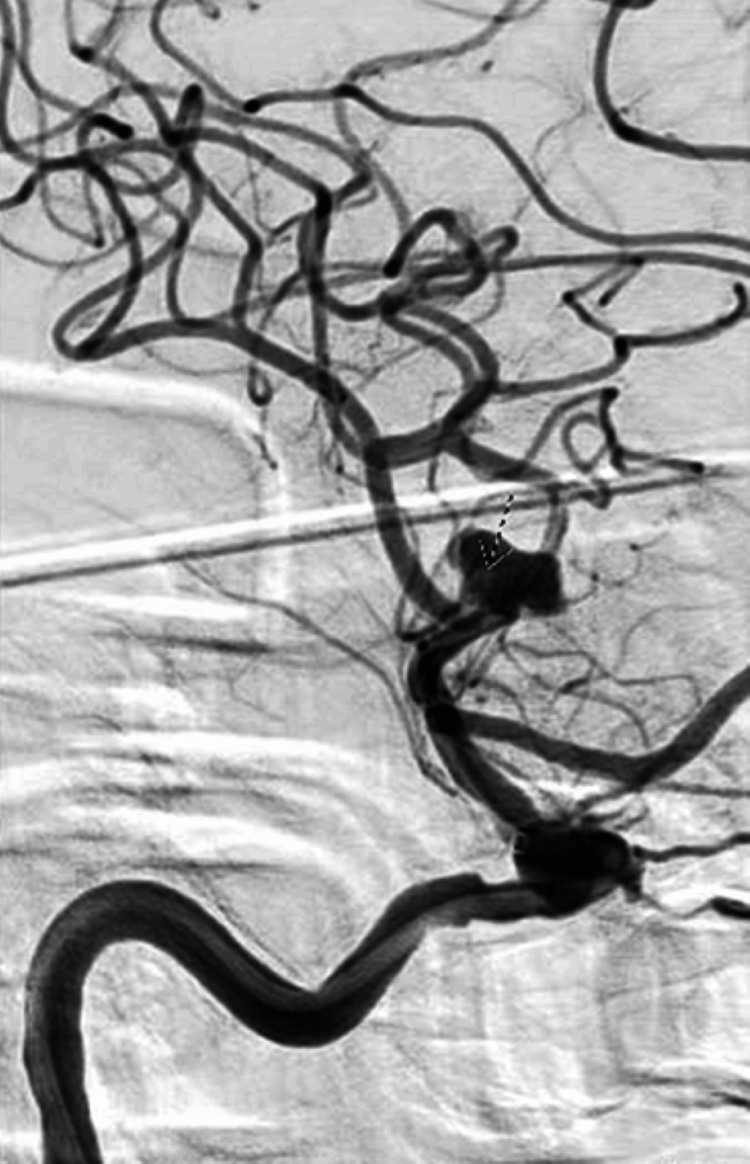
Cerebral angiography for a flow-diverting stent. The arrow demonstrates the defect consistent with intra-aneurysmal thrombus.

After multidisciplinary consensus (including neurosurgery, interventional neuroradiology, neurology, and neurocritical care), the lesion was treated with flow diversion to trap intra-aneurysmal thrombus, mitigate rupture risk, and enable safe initiation of dual antiplatelet therapy. Using a Phenom 021 microcatheter (Medtronic, Santa Rosa, CA) over a Synchro Standard guidewire (Stryker Neurovascular, Fremont, CA), the device was carefully navigated across the aneurysm into the superior division, avoiding contact with the filling defect. A FRED 21 (Terumo Neuro, Aliso Viejo, CA) 2.5 x 13 mm flow diverter was deployed from the superior division into the M1 segment (Figure 5). The procedure achieved good flow diversion with progressive stasis within the aneurysm sac and no intra-procedural complications.

**Figure 6:**
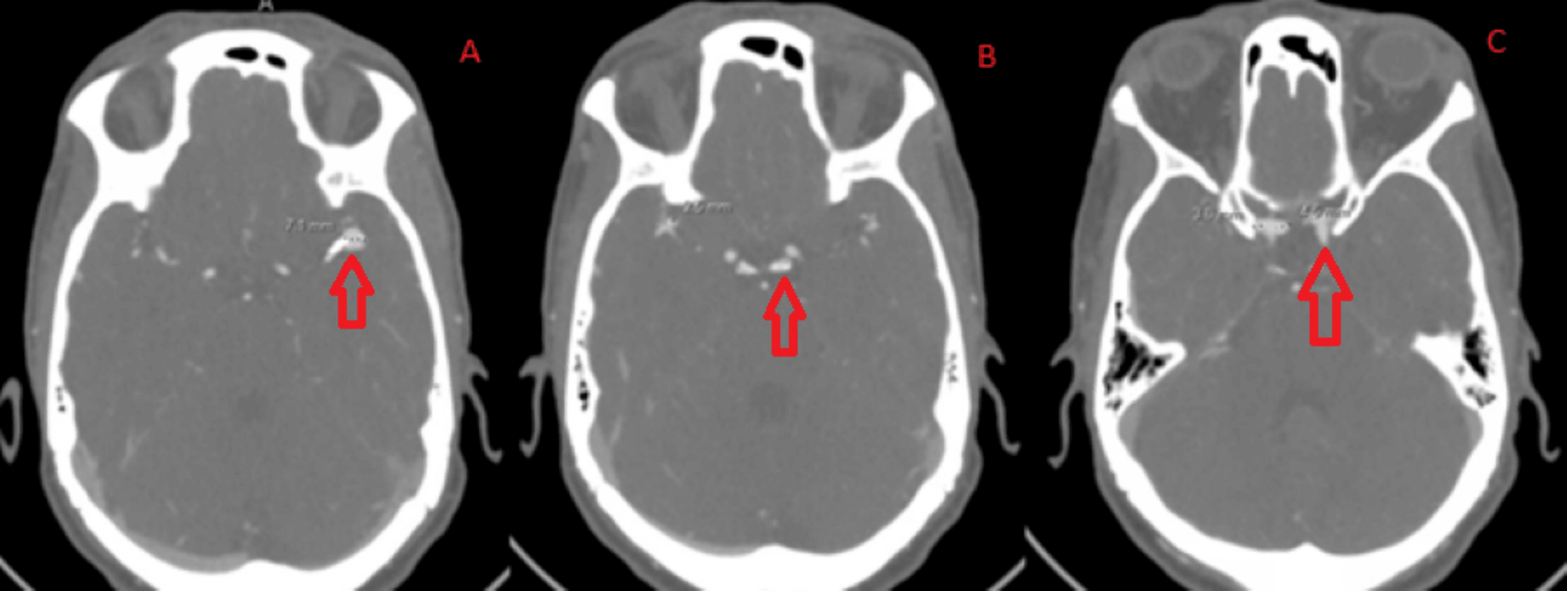
Follow-up computed tomography angiography two days after the procedure at high (A), middle (B), and deep (C) cross-sections.

Follow-up head CTA 48 hours later demonstrated a stable device position with preserved superior division flow and persistent aneurysm sac opacification (Figure 6). The patient recovered without new deficits and was discharged on postoperative day 4 on dual antiplatelet therapy and transitioned to aspirin monotherapy after six months. The patient refused to follow up in person, but a phone call follow-up at two years is reassuring that there have been no further neurological events.

## Discussion

Spontaneous thrombosis of intracranial aneurysms causing ischemic stroke is extremely rare. Our literature review did not identify previously reported instances of acute thrombosis occurring within an unruptured saccular MCA bifurcation aneurysm subsequently treated with endovascular flow diversion. This report highlights the diagnostic challenges and the therapeutic uncertainty with this presentation.

The patient had few conventional vascular risk factors aside from cigarette smoking, and the etiology of the acute thrombosis within an otherwise unremarkable, mid-sized MCA bifurcation aneurysm is uncertain. Aneurysm thrombosis in the setting of subarachnoid hemorrhage is more common, potentially due to overexuberant clot formation at the rupture site or later on due to vasospasm [5]. Although our patient presented without aneurysm rupture, we had to consider the possibility of a mural defect underlying the thrombus with potential for hemorrhage if there was a subsequent lysis of the clot.

Imaging played a central role in the diagnostic process. The initial CTA raised the possibility of a fusiform configuration; however, vessel wall MRI clarified the aneurysmal morphology and identified filling irregularities consistent with intra-aneurysmal thrombus. While vessel wall imaging has not yet been adopted universally, emerging evidence suggests that it can provide meaningful adjunctive information, particularly in differentiating aneurysmal subtypes and in identifying features of instability [6,7]. Enhancement patterns on vessel wall imaging have been associated with rupture risk, especially in small aneurysms (<7 mm) that are otherwise observed rather than treated [7]. Nevertheless, interpretation remains limited by heterogeneity in acquisition protocols and the absence of standardized criteria for wall enhancement. Further validation of objective, automated methods across institutions would facilitate broader clinical adoption.

From a therapeutic standpoint, the decision to employ a flow-diverting stent was guided by the dual need to prevent recurrent thromboembolism while simultaneously protecting against rupture. Although flow diversion is more commonly applied to wide-necked or fusiform aneurysms [8], its mechanism of inducing stasis and progressive thrombosis of the aneurysm sac made it a logical strategy in this setting. The major concern was the potential for mobilization of pre-existing thrombus during flow alteration. Initiation of dual antiplatelet therapy mitigated this risk, and in our patient, the approach was technically successful and clinically well tolerated.

## Conclusions

The management paradigm for unruptured, partially thrombosed bifurcation aneurysms presenting with ischemic stroke is not yet established. This case demonstrates that flow diversion with antiplatelet therapy can be a viable option, balancing the competing risks of recurrent embolization and aneurysmal rupture. However, further experience and systematic data collection are essential to better define the role of this strategy, ideally through collaborative multicenter registries.
